# 3-D-Druck-optimierte Anpassung eines Mittelgesichtsimplantats zur magnetgetragenen nasalen Epithesenversorgung

**DOI:** 10.1007/s00106-021-01100-6

**Published:** 2021-08-31

**Authors:** Christian Wrobel, Daniel Keppeler, Alexander C. Meyer

**Affiliations:** 1 Klinik für Hals-Nasen-Ohrenheilkunde, Universitätsmedizin Göttingen, Georg-August-Universität Göttingen, Robert-Koch-Str. 40, 37075 Göttingen, Deutschland; 2 Institut für Auditorische Neurowissenschaften, Universitätsmedizin Göttingen, 37075 Göttingen, Deutschland

**Keywords:** Titanimplantat, Karzinom der Nase, Plattenbasierte Ankersysteme, Faziale Rekonstruktion, Rhinektomie, Titanium implant, Nose carcinoma, Plate-based anchorage systems, Facial reconstruction, Rhinectomy

## Abstract

**Hintergrund:**

Plattenbasierte Ankersysteme zur fazialen Epithesenversorgung bieten gegenüber extraoralen Einzeltitanimplantaten Vorteile hinsichtlich einer flexibleren Wahl knöcherner Verankerungspunkte und höherer Stabilität. Nachteile werden in einer aufwendigen individuellen intraoperativen Anpassung der Plattensysteme am meist schlecht zugänglichen Knochen deutlich. Wir stellen eine Methode vor, diese Nachteile zu überwinden und die Vorteile plattenbasierter Systeme stärker auszuspielen.

**Methodik:**

Das knöcherne Mittegesicht eines Patienten mit erfolgter Rhinektomie bei Karzinom des Naseneingangs wurde anhand der präoperativen Computertomographie als virtuelles 3‑D-Modell rekonstruiert. Die verwendete Open-Source-Software (3-D-Sclicer) ermöglichte die einfache und schnelle Rekonstruktion sowie Anpassung zum Druck des 3‑D-Modells mittels transparenten Kunststoffs (MED610; stratasys Ltd., MN, USA).

**Ergebnisse:**

Die als Epithesenanker verwendete Titan-Brückenplatte (MEDICON) konnte am 3‑D-Druck des Mittelgesichts äußerst präzise vorangepasst werden. Wichtige anatomische Strukturen wurden geschont und die Verschraubungspunkte entsprechend der gegebenen Knochendicke gewählt. Die Implantation der vorangepassten Titanplatte erfolgte komplikationslos ohne weitere intraoperative Anpassungen.

**Schlussfolgerung:**

Die Voranpassung plattenbasierter Ankersysteme für faziale Epithesen am 3‑D-Druck des Mittelgesichts überwindet deren Nachteile einer aufwendigen ggf. unpräzisen intraoperativen individuellen Anpassung. Diese Methode spielt die Vorteile der besseren Kraftverteilung durch mehr mögliche Verschraubungen, auch in dünnerem Knochen, weiter aus und kann somit Implantatlockerungen vorbeugen. Zudem ermöglicht die Voranpassung am 3‑D-Modell die bessere Identifikation und Schonung wichtiger anatomischer Strukturen und spart Op.-Zeit ein.

## Abstract (en)

**Background:**

Plate-based anchorage systems for craniofacial prostheses offer advantages over extraoral solitary titanium implants in terms of the flexible choice of mounting points and higher stability. Disadvantages become apparent in the complex individual intraoperative adaptation of the plate-based systems to the usually poorly accessible bone. The current article presents a method to overcome these disadvantages and make greater use of the advantages of plate-based systems.

**Materials and methods:**

The bony midface of a patient who had undergone rhinectomy for cancer of the nasal entrance was reconstructed as a virtual 3D model based on preoperative CT. The open-source software (3D-Slicer) allowed easy and fast reconstruction as well as adaptation for 3D printing using transparent plastic (MED610; stratasys Ltd., MN, USA).

**Results:**

A titanium mini-plate (MEDICON) for anchoring the nasal prosthesis could be fitted extremely precisely on the midface 3D print. Important anatomical structures were spared, and screw placement was selected according to the individual bone thickness. Implantation of the in-advance fitted titanium plate was performed without complications and without further adjustments.

**Conclusion:**

In-advance fitting of plate-based systems for anchorage of craniofacial prostheses using 3D printing of the midface overcomes their disadvantages of time-consuming and possibly imprecise individual adaptation. This method further exploits the advantages of higher stability through more possible mounting points, even in thinner bone, to prevent loosening. In addition, in-advance fitting of titanium plates on the 3D model enables better identification and protection of important anatomical structures and shortens operative time.

Ursachen ausgedehnter Defekte der äußeren Nase können kongenitaler, traumatischer oder onkologisch-chirurgischer Natur sein. In der klinischen Praxis sind wir jedoch mit Abstand am häufigsten mit Folgezuständen nach partieller oder totaler Rhinektomie bei Malignomen der äußeren Nase konfrontiert [1–3]. Diese besitzen, da in der Regel hochgradig entstellend, erhebliche negative psychosoziale Auswirkungen auf die betroffenen Patienten [4]. Die chirurgische Rekonstruktion von Funktion und Ästhetik der äußeren Nase ist anspruchsvoll. Häufig sind mehrere aufeinander aufbauende Operationen notwendig, die auch die Transplantation von autologem Gewebe bedingen. Nichtsdestotrotz stellen moderne chirurgisch-rekonstruktive Techniken ihr Potenzial für hervorragende Ergebnisse regelmäßig unter Beweis [5]. Eine nicht unerhebliche Zahl an Patienten kommt jedoch für diese Versorgung aufgrund von Alter, Vorerkrankungen oder Compliance nicht infrage [6–10]. Für diese Gruppe stellt die epithetische Versorgung eine wichtige Alternative zur chirurgischen Rekonstruktion dar. Die Vorteile liegen hier in der deutlichen Reduktion von chirurgischer Invasivität und Aufwand, bei gleichzeitig guten Ergebnissen hinsichtlich Ästhetik und Funktion. Auch die Tumornachsorge profitiert von der regelmäßig guten Einsehbarkeit der ehemaligen Tumorregion [4, 11].

Die einfachste Form der Verankerung von Epithesen der äußeren Nase stellen Klebeverbindungen mit der Haut dar. Bei ungünstigem Defekt, starker Schweiß- oder Fettbildung kommt es bei dieser Methode jedoch leicht zu Lockerungen, sodass sich transkutane Titanimplantate als Alternative bewährt haben [12, 13]. Den Grundstein hierfür legten Brånemark, Albrektsson und Tjellström in den 1970er-Jahren mit permanenten extraoralen transkutanen Titanimplantaten, zunächst für knochenverankerte Hörgeräte und dann für die Verankerung von Epithesen des äußeren Ohrs [14–16]. Später erfolgte ihr Einsatz auch bei der epithetischen Versorgung maxillofazialer und orbitaler Defekte [12]. Als Verbindung zwischen Implantat und Epithese haben sich zunehmend magnetische Systeme durchgesetzt, welche einerseits eine hohe Haltekraft bieten, andererseits aber auch die einfache Handhabung und Reinigung von Epithese und Verankerung ermöglichen [17, 18]. Für die Herstellung nasaler Epithesen wird regelhaft Silikon als Material verwendet, welches gegenüber anderen Kunststoffen, Glas, Gummi, Porzellan, o. Ä. eine Reihe von Vorteilen aufweist: Es kann sehr genau der Form, Farbe und Oberflächenstruktur der Nase des Patienten angepasst werden, es ermöglicht äußerst dünne Ränder, die den Übergang zur gesunden Haut kaum erkennen lassen, es ist flexibel und passt sich auch der Körpertemperatur an, was den Tragekomfort erhöht [19].

Neben den beschriebenen extraoralen knochenverankerten Einzeltitanimplantaten stehen auch plattenbasierte Ankersysteme für die Epithesenversorgung zur Verfügung [19]. Diese Form der Verankerung bietet den Vorteil, die Verankerungspunkte im Knochen relativ frei, ggf. auch mit Abstand zu dem zu versorgenden Defekt zu wählen. Es bleibt jedoch eine operative Herausforderung, die Ankerplatte und deren Fixierung optimal sowohl an den tragenden Knochen der Maxilla anzupassen als auch eine problemlose spätere Anpassung der Epithese zu ermöglichen.

Wir stellen in dieser Arbeit eine Methode vor, diese Anpassung bereits präoperativ an einem mittels 3‑D-Druck erstellten Modell der Maxilla des Patienten exakt vorzunehmen. Hierdurch erhöht sich einerseits die Genauigkeit der Anpassung an den Oberkieferknochen bei andererseits deutlich verkürzter Operationszeit.

## Material und Methodik

**Figure 1:**
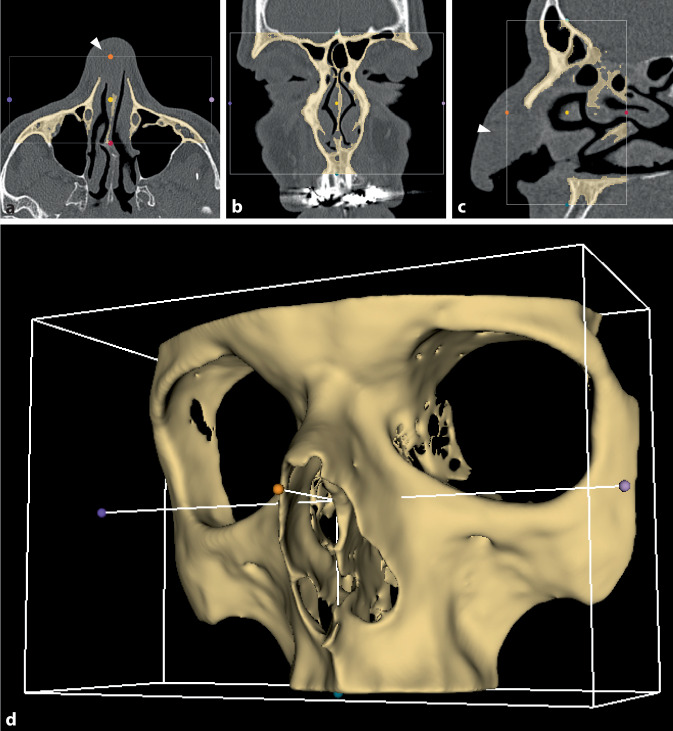

Die hier beschriebene Methode zur 3‑D-Druck-optimierten Anpassung eines magnettragenden Mittelgesichtsimplantats zur nasalen Epithesenversorgung wurde an einem 56-jährigen männlichen Patienten mit einem nasalen Defekt nach Resektion eines Plattenepithelkarzinoms des rechten Naseneingangs (TNM: pT2 nach Wang [20] pN0 cM0 L0 V0, Grading: G2) angewendet. Der Tumor umfasste rechtsbetont die Nasenspitze und infiltrierte das anteriore Nasenseptum sowie auch partiell den linken knöchernen Naseneingang (Abb. 1a,c, Pfeil). Es erfolgte zunächst die Tumorresektion im Sinne einer subtotalen Ablatio nasi mit Neck-Dissection beidseits.

Die DICOM-Daten (*Digital Imaging and Communications in Medicine*) der zum Staging durchgeführten Computertomographie (Knochenfenster ohne Kontrastmittel, 0,6 × 0,6 mm Pixelgröße) wurden in der Open-Source-Software 3‑D-Slicer zu einem 3‑D-Modell verarbeitet [21]. In einem ersten Schritt wurde das vordere Mittelgesicht als *Region of Interest *definiert und in dieser mittels automatisierter Schwellenwerterkennung von Grauwerten (Otsu-Methode [22]) der Knochen vom übrigen Gewebe segmentiert (Abb. 1a–c). Dies diente als Grundlage für die Erstellung eines virtuellen 3‑D-Modells des vorderen Mittelgesichts (Abb. 1d). Zur besseren Verarbeitung durch den 3‑D-Drucker wurden zudem automatisiert kleine (< 10.000 Voxel) freie Inseln entfernt. Für den 3‑D-Druck wurde der segmentierte Datensatz im STL-Format (STereoLithography, 3D Systems Inc., SC, USA) gespeichert.

**Figure 2:**
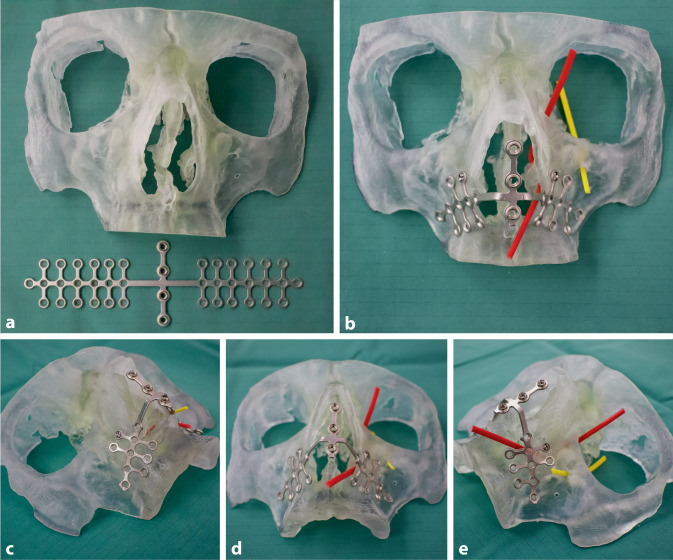

Eine weitere Optimierung des STL-Datensatzes für den 3‑D-Drucker erfolgte durch die frei verfügbare Software *Autodesk Meshmixer* (Autodesk Inc., CA, USA) mit den Werkzeugen *Inspektor* (Füllmodus, Schwellenwert: 0,1 mm) und *Materialstärkenprüfung*. Anschließend wurde das 3‑D-Modell des vorderen Mittelgesichts mit dem biokompatiblen, transparenten Material MED610 (stratasys, Ltd., MN, USA) an einem 3‑D-Drucker Typ stratasys Connex 3 Objet350 (stratasys Ltd., MN, USA) gedruckt und per Wasserstrahl vom Stützmaterial getrennt (Abb. 2a).

Als magnettragender Epithesenanker wurde eine Titan-MINI-Brückenplatte (1 mm Plattenstärke, 115 mm Länge, Art.-Nr. 68.80.35) aus dem Epiplating MINI-Plattensystem der Fa. Medicon (MEDICON eG, Tuttlingen) verwendet, welche mit entsprechendem Instrumentarium angepasst wurde.

## Ergebnisse

Das Verarbeiten der DICOM-Daten zum druckbaren virtuellen 3‑D-Modell des Mittelgesichts nach der oben beschrieben Methode nahm nicht mehr als 30 min in Anspruch. Die Materialkosten für den 3‑D-Druck betrugen ca. 130 €, wobei die Kosten für Anschaffung und Wartung des Druckers nicht berücksichtigt sind. Kosten für einen 3‑D-Druck aus gleichem oder äquivalentem Material hätten bei verschiedenen externen Dienstleistern 200–300 € betragen.

Die Anpassung der Titan-Brückenplatte – Kürzen, Biegen und Abschleifen – erfolgte unter nichtsterilen Bedingungen am 3‑D-Modell des vorderen Mittelgesichts (Abb. 2b–e) und nahm insgesamt 40 min in Anspruch. Das transparente Material ermöglichte die genaue Lokalisation von Ductus nasolacrimalis und Foramen infraorbitale, Canalis infraorbitalis (Abb. 2b–e, rote bzw. gelbe Markierung) sowie den Zahnwurzeln und somit die Platzierung der Schrauben unter Schonung der genannten Strukturen. Die Verwendung eines 3‑D-Modells des vorderen knöchernen Mittelgesichts ermöglichte ein äußerst präzises Anpassen der Titan-Brückenplatte ohne Druck oder Zug durch Muskulatur, Bindegewebe oder Haut. Nach Anpassung wurden Mittelgesichtsimplantat und entsprechendes Instrumentarium normal dampfsterilisiert.

**Figure 3:**
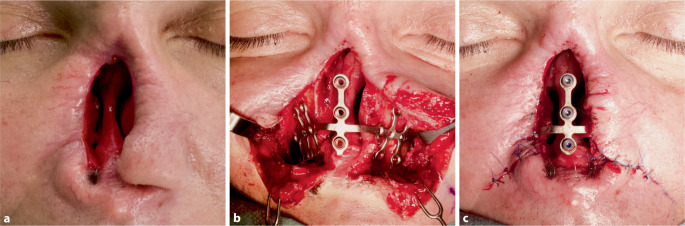

Die Implantation der Titan-Brückenplatte erfolgte 3 Monate nach der Tumorresektion im Sinne einer subtotalen Ablatio nasi (Abb. 3a). Die nötige anteriore Fläche der Maxilla wurde freigelegt und die angepasste Titan-Brückenplatte konnte ohne weitere Modifikation wie geplant mittels vier 5,5 mm Titanschrauben je Seite subperiostal fixiert werden (Abb. 3b). Abschließend wurde das Weichgewebe über dem Implantat vernäht (Abb. 3c). Insgesamt betrug die Eingriffszeit etwa eine Stunde.

## Diskussion

Plattenbasierte Ankersysteme zur Epithesenversorgung werden im deutschsprachigen Raum neben den extraoralen knochenverankerten Einzeltitanimplantaten häufig verwendet [17]. Zumeist, wie auch in dieser Arbeit, kommt das Medicon Titan Epiplating System (MEDICON eG, Tuttlingen) zum Einsatz, welches spezielle aurikuläre, nasale und orbitale sowie universale Titanplatten zur subperiostalen Implantation bereitstellt. Durch multiple Verschraubungen verteilen sich die entstehenden Kräfte besser, die einzelnen Knochenschrauben können kleiner ausfallen und damit auch in dünnerem Knochen sicher fixiert werden. Zudem besteht ein höherer Widerstand gegenüber Drehkräften, welche zu einer Lockerung des Implantats führen können [19]. Neben den genannten Vorteilen besitzen plattenbasierte Systeme gegenüber Einzelimplantaten auch Nachteile. So sind die intraoperative individuelle Anpassung und Platzierung aufwendiger und anspruchsvoller.

Die individuelle Anpassung der Titanplatten an die knöcherne Anatomie des Patienten kostet intraoperativ vor allem Zeit. Durch die zeitliche Vorverlagerung der Plattenanpassung mittels 3‑D-Modell des Mittelgesichtsknochens kann Op.-Zeit – die Plattenanpassung am 3‑D-Druck nahm ca. 40 min in Anspruch – eingespart werden. Somit können die mit längerer Op.- und Narkose-Zeit verbundenen Komplikationen reduziert werden [23, 24]. Verschiedene Verfahren der Anpassung von Titanimplantaten am gedruckten 3‑D-Modell sind in der Chirurgie – beispielsweise bei der Versorgung komplexer Frakturen – etabliert. Sie haben hierbei ihr Einsparpotential hinsichtlich Op.- und Narkosezeit unter Beweis gestellt [25]. Wir beschreiben hier erstmalig die Verwendung dieser Technik im Rahmen der implantatgetragenen epithetischen Versorgung.

Lockerungen knochenverankerter Einzel-Titan-Implantate zeigen sich in der nasalen und orbitalen Region signifikant häufiger als im Bereich des Mastoids, was auf den deutlich dünneren Knochen in diesen Regionen zurückgeführt wird [26]. Eine Radiotherapie erhöht die Wahrscheinlichkeit einer Implantatlockerung [26]. Hier können plattenbasierte Ankersysteme Ihre Vorteile ausspielen: Die publizierten retrospektiven Untersuchungen nach nasaler Epithesenversorgung unter Verwendung des Medicon Epiplating Systems zeigen eine Implantat-Überlebensrate von 82–96,2 % im untersuchten Follow-up-Zeitraum von mindestens 2 Jahren [27–29]. Die hier vorgestellte Methode bietet durch die präoperative Anpassung der Titanplatten am transparenten 3‑D-Druck des knöchernen Mittelgesichts die Möglichkeit der optimalen Platzierung der Verankerungsschrauben in ausreichend stabilem Knochen. Dies sollte die Wahrscheinlichkeit von Implantatlockerungen selbst in bestrahltem Knochen weiter reduzieren. Zudem können funktionell wichtige Strukturen wie Ductus nasolacrimalis und N. infraorbitalis problemlos identifiziert und geschont werden. Die Abbildung des Originals durch ein auf der Basis von DICOM-Daten rekonstruiertes 3‑D-Modell von knöchernen Strukturen – aufgearbeitet und hergestellt mit äquivalenten Methoden (CT-Auflösung, virtuelle 3‑D-Rekonstruktion und 3‑D-Drucktechnik) – ist mit deutlich unter einem Millimeter sehr genau [30]. Eine auf dem aktuellen Standard durchgeführtes präoperative CT zum Staging ist für die hier vorgestellte Methode in der Genauigkeit ausreichend, eine zusätzliche Strahlenbelastung kommt somit nicht auf.

Die zeitlich vorverlagerte Anpassung der Titanankerplatten mit der hier vorgestellten Methode hilft darüber hinaus, den operativen Zugang minimal zu gestalten und damit einer Narbenbildung im Gesicht entgegenzuwirken. Wir erwarten zudem bei geringerer Invasivität eine Reduktion von postoperativen Schwellungen und Hämatombildungen und des Risikos für Infektionen und Wundheilungsstörungen, welche nach Plattenimplantation auftreten können [27–29]. Weiterhin wäre es möglich, die Implantation eines Titanankers in gleicher Sitzung mit der Tumorresektion durchzuführen. Gerade wenn eine adjuvante Strahlentherapie der Tumorregion erforderlich erscheint, kann bei diesem Vorgehen eine bessere Osseointegration erwartet werden. [29, 31].

## Fazit für die Praxis

- Plattenbasierte Ankersysteme zur fazialen Epithesenversorgung bieten Vorteile hinsichtlich Flexibilität und Stabilität gegenüber knochenverankerten Einzeltitanimplantaten.
- Die präoperative Anpassung der Titanplatten am 3‑D-Druck vermeidet deren Nachteile hinsichtlich aufwendiger intraoperativer Anpassung und verringert damit die Op.-Zeit und Invasivität des Eingriffs.
- Die hier vorgestellte Methode erlaubt eine äußerst präzise Positionierung der Verschraubung unter Schonung wichtiger anatomischer Strukturen und Einbeziehung der Knochendicke am 3‑D-Modell.

## References

[1] Stanley RJ, Olsen KD (1988). Rhinectomy for malignant disease. A 20-year experience. Arch Otolaryngol Head Neck Surg.

[2] Johnson JT (1993). Management of advanced cancers of the external nose. Oncology (Williston Park).

[3] Chipp E, Prinsloo D, Rayatt S (2011). Rhinectomy for the management of nasal malignancies. J Laryngol Otol.

[4] Faris C (2020). Health utility of rhinectomy, surgical nasal reconstruction, and prosthetic rehabilitation. Laryngoscope.

[5] Phillips TJ (2019). Total nasal reconstruction: a review of the past and present, with a peak into the future. Curr Opin Otolaryngol Head Neck Surg.

[6] Menick FJ (2010). Nasal reconstruction. Plast Reconstr Surg.

[7] Menick FJ (2009). Nasal reconstruction with a forehead flap. Clin Plast Surg.

[8] Thornton JF, Griffin JR, Constantine FC (2008). Nasal reconstruction: an overview and nuances. Semin Plast Surg.

[9] Burget GC, Walton RL (2007). Optimal use of microvascular free flaps, cartilage grafts, and a paramedian forehead flap for aesthetic reconstruction of the nose and adjacent facial units. Plast Reconstr Surg.

[10] Rohrich RJ, Griffin JR, Ansari M, Beran SJ, Potter JK (2004). Nasal reconstruction—Beyond aesthetic subunits: a 15-year review of 1334 cases. Plast Reconstr Surg.

[11] Korfage A, Raghoebar GM, Noorda WD, Plaat BE, Vissink A, Visser A (2016). Recommendations for implant-retained nasal prostheses after ablative tumor surgery: minimal surgical aftercare, high implant survival, and satisfied patients. Head Neck.

[12] Ariani N (2013). Current state of craniofacial prosthetic rehabilitation. Int J Prosthodont.

[13] Leonardi A, Buonaccorsi S, Pellacchia V, Moricca LM, Indrizzi E, Fini G (2008). Maxillofacial prosthetic rehabilitation using extraoral implants. J Craniofac Surg.

[14] Tjellström A, Lindström J, Hallén O, Albrektsson T, Brånemark PI (1981). Osseointegrated titanium implants in the temporal bone. A clinical study on bone-anchored hearing aids. Am J Otol.

[15] Tjellström A (1981). The bone-anchored auricular episthesis. Laryngoscope.

[16] Brånemark PI, Albrektsson T (1982). Titanium implants permanently penetrating human skin. Scand J Plast Reconstr Surg.

[17] Thiele OC (2015). The current state of facial prosthetics—A multicenter analysis. J Craniomaxillofac Surg.

[18] Alvi R, McPhail J, Hancock K (2002). Closed-field titanium magnets for the retention of complex craniofacial prostheses. Br J Plast Surg.

[19] Federspil PA (2009). Implant-retained craniofacial prostheses for facial defects. GMS Curr Top Otorhinolaryngol Head Neck Surg.

[20] Wang CC (1976). Treatment of carcinoma of the nasal vestibule by irradiation. Cancer.

[21] Fedorov A (2012). 3D slicer as an image computing platform for the quantitative imaging network. Magn Reson Imaging.

[22] Otsu N (1979). A threshold selection method from Gray-level histograms. IEEE Trans Syst Man Cybern.

[23] Kim BD, Hsu WK, De Oliveira GS, Saha S, Kim JYS (2014). Operative duration as an independent risk factor for postoperative complications in single-level lumbar fusion: an analysis of 4588 surgical cases. Spine (Phila Pa 1976).

[24] Procter LD, Davenport DL, Bernard AC, Zwischenberger JB (2010). General surgical operative duration is associated with increased risk-adjusted infectious complication rates and length of hospital stay. J Am Coll Surg.

[25] Hoang D, Perrault D, Stevanovic M, Ghiassi A (2016). Surgical applications of three-dimensional printing: a review of the current literature & how to get started. Ann Transl Med.

[26] Chrcanovic BR, Nilsson J, Thor A (2016). Survival and complications of implants to support craniofacial prosthesis: a systematic review. J Craniomaxillofac Surg.

[27] Sandner A, Bloching M (2009). Retrospective analysis of titanium plate-retained prostheses placed after total rhinectomy. Int J Oral Maxillofac Implants.

[28] Lünenbürger H, Roknic N, Klein M, Wermker K (2016). Treatment outcome of the transfacial titanium epiplating system for total nasal defects. Plast Reconstr Surg.

[29] Papaspyrou G, Schick B, Schneider M, Al Kadah B (2017). Epithetic nasal reconstruction for nasal carcinoma: retrospective analysis on 22 patients. Eur Arch Otorhinolaryngol.

[30] George E, Liacouras P, Rybicki FJ, Mitsouras D (2017). Measuring and establishing the accuracy and reproducibility of 3D printed medical models. Radiographics.

[31] Ozen J, Dirican B, Oysul K, Beyzadeoglu M, Ucok O, Beydemir B (2005). Dosimetric evaluation of the effect of dental implants in head and neck radiotherapy. Oral Surg Oral Med Oral Pathol Oral Radiol Endod.

